# Advancing integrated care and its evaluation by means of a universal typology

**DOI:** 10.1177/2053434517705732

**Published:** 2017-04-27

**Authors:** Loraine Busetto, Katrien Luijkx, Hubertus Johannes Maria Vrijhoef

**Affiliations:** 1 Tranzo Scientific Center for Care and Welfare, Tilburg University, the Netherlands; 2 Panaxea B.V., Amsterdam, The Netherlands; 3 Maastricht University Medical Center, the Netherlands; 4 Vrije Universiteit Brussel, Belgium

**Keywords:** Integrated care, Triple Aim, typology

## Abstract

Health systems around the globe implement integrated care interventions to address the Triple Aim of simultaneously improving population health, patient experiences and cost-efficiency. However, the underlying definitions and conceptualisations of integrated care often differ considerably, which makes uniform measurement and comparison difficult. Rather than agreeing on one definition of integrated care, we argue that a universal typology of integrated care interventions should be developed to enable the comparison of interventions that are based on different understandings of integrated care. This universal typology should combine rankable and intangible components with unrankable and tangible sub-components, and be conceptually sound and flexible. The content of the typology should be developed by an international consortium of relevant stakeholders.

## Introduction

Many health systems have endorsed integrated care in response to an increasing demand for complex long-term care, and there seems to be an almost universal consensus on expecting that integrated care will simultaneously contribute to improved population health, patient experiences and cost-efficiency, that is, the Triple Aim.^1,2^ However, there is a huge variation in opinions about what integrated care actually is or should be, often depending on the specific research focus, cultural or professional backgrounds, or the timing of the research.^3^ For example, in the United Kingdom the so-called integrated care pilots concern the integration of health and social care services,^4^ whereas the so-called vanguard sites target integrated primary and acute care systems, enhanced health in care homes and multispecialty community provider systems.^5^ Other examples include population health management pilots in the Netherlands,^6^ selective health insurance contracts for providers from different sectors in the German health system^7^ and next generation accountable care organisations in the United States.^8^

For over a decade, the academic community has been trying to resolve this issue by developing different definitions of integrated care and striving for agreement on “the best” of these.^9–12^ We argue that rather than aiming for agreement on one definition of integrated care, which is unlikely, we are in need of a universal typology that allows for the description of all of these different interventions that are considered integrated care by different stakeholder groups, while respecting local colour. We believe this to be a necessary tool to make different integrated care interventions and their components observable, identifiable, measurable and therefore comparable, which is a necessary step to move integrated care and its evaluation forward. Additionally, such a typology would make it possible to distinguish the mechanisms of integrated care from their context on the one hand and their outcomes on the other hand. This distinction was originally described in the Context–Mechanisms–Outcomes (CMO) Model^13^ and recently adapted to integrated care in the COMIC model for studying the context, outcomes and mechanisms of integrated care interventions.^14^ In this model, mechanisms refer to the type of integrated care intervention, context to the setting in which mechanisms are brought into practice and outcomes as effects triggered by mechanism and context.

## Existing typologies of integrated care

Many taxonomies or typologies of integrated care are already in existence, distinguishing for example between different types, forms or models of integrated care.^10,15–23^ These typologies can be typologised themselves according to the rankability and tangibility of their components. Tangible components are empirically measurable, as opposed to intangible components which are theory-based and non-empirical. As a general rule, tangible components can be observed or implemented in practice (e.g. multidisciplinary protocols), whereas intangible components are often abstract concepts used to label groups of tangible components that need to be operationalised before they can be implemented in practice (e.g. system integration). Rankable components can be ranked in a meaningful way (e.g. from less integration to more integration or from the micro to the macro level), as opposed to unrankable components for which this is not possible (e.g. vertical and horizontal integration). For example, Leutz described an intangible and rankable typology by describing the three theoretical concepts linkage, coordination and full integration, representing an increasing degree of integration along a continuum.^19^ The distinction between real and virtual integration^21^ represents an example of a typology whose components are intangible and unrankable.

Kodner and Spreeuwenberg^12^ and Valentijn et al.^24^ developed typologies that combine intangible and rankable components with tangible and unrankable sub-components. Specifically, Kodner and Spreeuwenberg define five intangible domains (funding, administrative, organisational, service delivery, clinical) that are rankable from the macro to the micro level. At each of these domains, tangible, unrankable methods and tools can occur, including, for example, the use of shared clinical records or common decision support tools (i.e. practice guidelines and protocols).^12^ The typology proposed by Valentijn et al. specifies six intangible dimensions of integration (clinical, professional, organisational, system, functional and normative integration) and three rankable levels (macro, meso, micro) around which these dimensions of integrated care are structured. The typology also has unrankable aspects because functional and normative integration operate at all three of these levels simultaneously. The six dimensions consist of tangible and unrankable sub-components including, for example, inter-professional education or individual multidisciplinary care plans.^24^

Using a specific typology generally reflects a specific understanding of integrated care as well as an intention to describe an intervention that fits this understanding. For example, if we want to compare whether one health system is less or more integrated than another, we would choose a typology that allows for rankability. In that case, a distinction between linkage, coordination and full integration would be more useful than one between horizontal and vertical integration. If we want to compare a national bundled payment system to the cooperation between two health care provider organisations, and to multidisciplinary geriatric assessments at a hospital ward, we need a typology that at least describes the system, inter-organisational and intra-organisational level. However, if we want to compare three different multidisciplinary geriatric assessment interventions to each other, the distinction between the system, inter-organisational and intra-organisational level would be less useful because all three interventions act at the same level. In this case, we would need a more detailed typology for the intra-organisational level that would let us describe more tangible components such as whether standardised communication protocols or single access points to care have been used. To reiterate, all these examples refer to comparing the mechanisms of integrated care, that is, the integrated care intervention itself, and not yet to the context in which the intervention is implemented or to the outcomes to which the intervention may contribute.

## A universal typology of integrated care

These considerations provide insights into the usability of a specific typology for a specific type of intervention or evaluation purpose. However, if we choose typologies according to the type of intervention we want to describe, we cannot compare or aggregate findings from studies describing different types of integrated care, precisely because they use different typologies. As with the discussion about the definition of integrated care, choosing ‘the best’ of existing typologies is not worthwhile, not the least because the former choice will probably inform the latter. We therefore aim for a universal typology of integrated care interventions, whose main strength and advantage lies in the fact that it enables the description of all of the different types of integrated care interventions (i.e. mechanisms) described above. Such a universal typology should have the following attributes:

First, its main components should be rankable from the macro (system or regulatory) to the micro (person at risk of or with illness) level, in order to have a meaningful ordering principle that reflects the level at which an intervention is implemented. As mentioned above, it is also possible for a typology to be rankable from less to more integration, but this option should be explored with caution, given that the typology should enable the comparison of a diverse set of interventions, for which more or less integration might hold different meanings. Second, these main components should be intangible. Intangible typologies tend to be more encompassing because they can cover all categories that are theoretically relevant instead of only those that can be or were observed in practice. For example, it is apparent that when one describes horizontal integration, there should also be the conceptual opposite, namely vertical integration, which is not as apparent for non-conceptual examples such as multidisciplinary teams or feedback. Moreover, intangible components can be theory-based and therefore make a typology more theoretically robust. For example, components could be based on the Chronic Care Model, which specifies areas for improvement for chronic illness care.^25^ Third, the rankable and intangible main components should be operationalised into or include examples of tangible sub-components or sub-categories as this makes it easier to apply the typology to practice examples of integrated care interventions. For example, it is easier to determine whether an intervention includes evidence-based guidelines than whether it concerns functional integration. Fourth, these sub-components should be unrankable because otherwise it would not be possible to list all the tangible examples observed in practice. Fifth, the typology should be conceptually sound, that is, it should only address the *mechanisms* of the integrated care intervention and not include *context* factors or intended *outcomes* as intervention components.^14^ For example, ‘client satisfaction’ or ‘reliable behaviour’ might be goals or factors that, if present, would facilitate implementation, but they do not constitute specific activities that are part of an intervention. If we want to conduct comprehensive evaluations that take into account how the context in which an intervention is implemented affects the outcomes achieved, we need to be clear about what is what. Sixth, the typology should be flexible enough to allow for new (sub-) components to be added in case new approaches or technologies are developed.

## Moving forward

These characteristics mainly pertain to the form of the typology. The content of the typology should be developed and validated in an international effort spanning different countries, cultures, health systems, professions and life experiences. A typology in itself is not an endpoint, but should be incorporated into more appropriate and comprehensive research designs which take into account the context of an intervention and its influence on outcomes achieved, so as to arrive at targeted recommendations for improvement, that will hopefully indeed contribute to improved population health, patient experiences and cost-efficiency. Given the early stage of large-scale evaluations of integrated care in various countries, we believe that this is the moment to join forces.
